# Investigating the Activities of *CAF20* and *ECM32* in the Regulation of *PGM2* mRNA Translation

**DOI:** 10.3390/biology13110884

**Published:** 2024-10-30

**Authors:** Mustafa Al-gafari, Sasi Kumar Jagadeesan, Thomas David Daniel Kazmirchuk, Sarah Takallou, Jiashu Wang, Maryam Hajikarimlou, Nishka Beersing Ramessur, Waleed Darwish, Calvin Bradbury-Jost, Houman Moteshareie, Kamaledin B. Said, Bahram Samanfar, Ashkan Golshani

**Affiliations:** 1 Department of Biology, Carleton University, Ottawa, ON K1S 5B6, Canada; mustafaalgafari@cmail.carleton.ca (M.A.-g.); sasikumarjagadeesan@cmail.carleton.ca (S.K.J.); tomkazmirchuk@cmail.carleton.ca (T.D.D.K.); saratakalloo@cmail.carleton.ca (S.T.); jiashuwang@cmail.carleton.ca (J.W.); maryamhajikarimlou@cmail.carleton.ca (M.H.); nishkabeersingramess@cmail.carleton.ca (N.B.R.); waleeddarwish@cmail.carleton.ca (W.D.); calvinbradburyjost@cmail.carleton.ca (C.B.-J.); kbs.mohamed@uoh.edu.sa (K.B.S.);; 2 Ottawa Institute of Systems Biology, University of Ottawa, Ottawa, ON K1N 6N5, Canada; houmanmoteshareie@cmail.carleton.ca; 3 Healthy Environments and Consumer Safety Branch, Health Canada, Ottawa, ON K1A 0K9, Canada; 4 Department of Pathology and Microbiology, College of Medicine, University of Hail, Hail P.O. Box 2240, Saudi Arabia; 5 Agriculture and Agri-Food Canada, Ottawa Research and Development Centre (ORDC), Ottawa, ON K1A 0C6, Canada

**Keywords:** functional genomics, lithium chloride, mRNA helicase, yeast, translation regulation, structured mRNA, translation, genetic interactions, sensitivity

## Abstract

Translation is a fundamental process in biology, and understanding its mechanisms is crucial to comprehending cellular functions and diseases. The regulation of this process is closely linked to the structure of mRNA, as these regions prove vital to modulating translation efficiency and control. Thus, identifying and investigating these fundamental factors that influence the processing and unwinding of structured mRNAs would be of interest due to the widespread impact in various fields of biology. To this end, we employed a computational approach and identified genes that may be involved in the translation of structured mRNAs. The approach is based on the enrichment of interactions and co-expression of genes with those that are known to influence translation and helicase activity. The in silico prediction found *CAF20* and *ECM32* to be highly ranked candidates that may play a role in unwinding mRNA. The activities of neither *CAF20* nor *ECM32* have previously been linked to the translation of *PGM2* mRNA or other structured mRNAs. Our follow-up investigations with these two genes provided evidence of their participation in the translation of *PGM2* mRNA and several other synthetic structured mRNAs.

## Simple Summary

In this study, we employ computational methods to identify genes that may influence the translation of structured mRNAs, namely *PGM2* and other synthetic mRNAs. We found two candidates, *CAF20* and *ECM32* that when deleted affect the translation of *PGM2* and synthetic mRNAs with structured 5’untranslated regions. These findings suggest that *CAF20* and *ECM32* have some involvement in the translation of structured mRNAs, expanding our understanding of mechanisms that influence gene regulation.

## 1. Introduction

Classical genetics, which focuses on a limited number of mutant phenotypes, has been a common approach in understanding a gene’s function; however, the field of genomics has been rapidly advancing [1]. Functional genomics approaches, including computational methods, have become increasingly utilized to attribute novel functions to genes involved in different biological processes in a quicker and more comprehensive manner [2,3,4]. These approaches can leverage existing datasets, such as protein–protein interactions (PPIs), genetic interactions (GI), and gene co-expression (GE) [5], to reveal functional connections for different genes. In doing so, our analysis can potentially identify novel gene functions. In this context, the baker’s yeast *Saccharomyces cerevisiae* is the model organism of choice to study novel eukaryotic gene functions.

PPI databases provide a wealth of information on the physical interactions between different proteins. High-throughput techniques such as yeast two-hybrid assays and tandem affinity tagging/purification have been instrumental in detecting new PPIs in various organisms [6]. Additionally, bioinformatics approaches and computational advancements have led to the formation of various databases, including Pfam [7] and DOMINE [8], which may represent a more comprehensive taxonomy of protein families, domains, and PPIs. Other popular PPI prediction engines (such as the Protein Interaction Prediction Engine [9]) utilize PPI databases (such as STRING [10]) to model novel PPIs and hence can predict novel gene functions [11,12].

Similarly, GI evidence can reveal functional interactions between genes and various cellular pathways. BioGRID [13] is an open-access database that focuses on GI data, along with other interactions, and facilitates additional gene function screenings. Cell Map [11] acts as a central repository for storing and interpreting information on quantitative genetic interactions. GI studies have revealed several novel gene functions in *E. coli* [14], yeast [15], and human cells [16].

Gene co-expression networks can also provide insights into the functional connection of genes and their phenotypes. A network for gene co-expression can be established by considering whether pairs or sets of genes demonstrate similar patterns of expression under varying conditions. For example, the Serial Pattern of Expression Levels Locator (SPELL) tool allows a collection of genes to be used as a query to generate an output list of genes of related co-expression profiles suggesting related functions [17]. Although the ability to anticipate new gene functions is limited by these computational approaches, they are invaluable for identifying potential targets for further experimental validation.

Translation control is a key step in the process of gene expression. Several genes have been recently identified to specifically regulate the translation of several structured mRNAs, including *PGM2* [18,19,20,21]. In light of the COVID-19 pandemic, the regulation of structured mRNA translation has received increased attention. SARS-CoV-2 mRNAs carry a specific structure in their 5′ UTR that mediates the translation of these mRNAs [22]. Similarly, the growing use of mRNA vaccines highlights the need to further study and elucidate the molecular factors that affect the efficient translation of these mRNA molecules [23]. Previous research on *PGM2* mRNA indicates that the translational control of this structured mRNA appears to be significantly more intricate than originally believed, with multiple protein factors affecting it [20,21,24]. The gene list that regulates the translation of structured mRNAs like *PGM2* is increasing, suggesting that other variables may affect this process [20,21,24,25,26]. 

In the current study, we aim to identify novel yeast gene functions involved in the regulation of gene expression by combining experimental verification with a computation-based functional genomics approach. Specifically, we focus on genes that regulate the mRNA translation of certain mRNAs that carry structured 5′ Untranslated Regions (UTRs). Our computational predictions are based on gene associations at the levels of PPI, GI, and GE networks in yeast. We follow up with the experimental validation of the activity of *CAF20*, thought to repress cap-dependent translation [27], and *ECM32*, a DNA helicase with reported activity in translation termination [28], in relation to their influence on the translation of certain structured mRNAs.

## 2. Materials and Methods

### 2.1. Computational Gene Function Prediction

**Figure 1:**
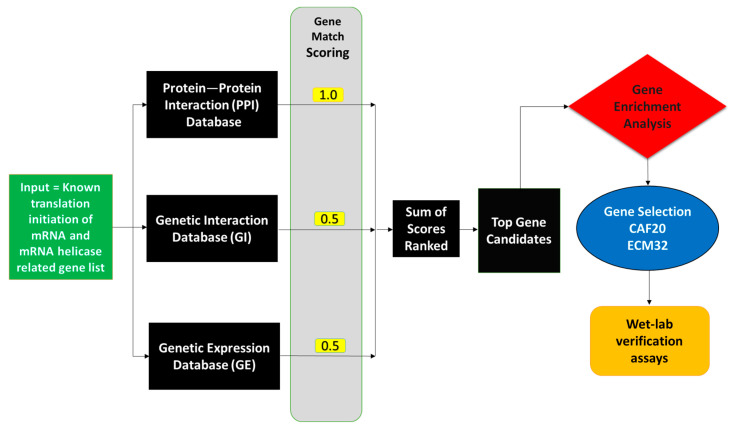
Schematic diagram of computational screening to identify genes involved in the translation of structured mRNAs. An input list of 71 genes (Table S1) involved in translation and helicase activity were evaluated for their reported PPIs, GIs, and GE. Each match within the PPI dataset was given a score of 1, and each match within the GE and GI datasets were scored as 0.5. Among the high-scoring gene candidates, *CAF20* and *ECM32* were the top two genes with no previously published direct functional links to the translation of structured mRNAs. These two genes were subjected to follow-up investigation.

We first established a network to identify candidate genes involved in translation initiation by using *S. cerevisiae* databases. This network was based on three criteria: PPIs, GIs, and GE (Figure 1). An input list of 71 genes that were previously associated with translation initiation and mRNA helicase activity in *S. cerevisiae* was utilized for gene selection [20,21,24,29,30]. To identify key interactors in the input list of genes, we utilized STRING with a confidence threshold value ≥ 0.7 for PPI analysis [31]. The active interaction sources used with STRING were Experiments, Database, Text mining, Co-expression, Neighborhood, Gene fusion, and Co-occurrence. For GI analysis, we used CellMap [32], while SPELL was employed for GE analysis [17]. Interactions and co-expression of genes in the input list were identified by using CellMap and SPELL, respectively. A candidate gene set was then selected based on the occurrence of genes in the PPI, GI, and GE datasets. A score of 1 was given for each gene match in the PPI dataset, 0.5 for each match in the GI dataset, and 0.5 for each match in the GE dataset, giving greater weight to protein interactions than genetic interactions, as is in [33]. Rankings were derived from the total scores for each gene. The gene candidate list threshold was a total score of 15 with genes scoring below this being dismissed, as the number of candidates was too large to be reliable. The top two candidates on this list were *CAF20* and *ECM32*, with scores of 47.5 and 47, respectively. Both candidates have no established relation to mRNA unwinding. In order to elucidate their functions in the translation of structured *PGM2* and other 5′ UTR constructs, these two genes were chosen for additional experimental validation.

### 2.2. Strains and Plasmids

The study utilized the MAT “α” mating strain Y7092 can1∆::STE2pr-Sp_his5 lyp1∆ man his3∆1 leu21∆0 ura3∆0 met15∆0 and the MAT “a” mating strain Y7092 Y4741 orf∆::KanMAX4 his3∆1 leu2∆0 met15∆0 ura3∆0. The yeast knockout collection provided the non-essential gene deletion mutant strains needed for Synthetic Genetic Analysis (SGA) [34]. Following the lithium acetate process, homologous recombination and PCR were used to create gene knockout strains, in accordance with previous methodology [35]. The overexpression plasmids of the gene candidates were obtained by using the yeast overexpression plasmid library [30]. Thermofisher^®^’s (Ottawa, Canada) Yeast GFP Clone Collection was used to create the PGM2p-GFP fusion strain needed for Western blot and qRT-PCR. To validate the overexpression plasmid, PCR analyses and chemical sensitivity were used. The p416 control plasmid carried no secondary structure in the 5′ UTR and contained a *LacZ* expression cassette that was controlled by the galactose (gal) promoter. *PGM2*, HIV1 (*TAR*), 2-hair, FOAP-11 (*RTN*), and BCL-2 (*Bcell*) are hairpin inserts designed and then cloned upstream of the *LacZ* mRNA of the p416 expression vector using the XbaI restriction site between the gal promoter and the *β-galactosidase* reporter gene (*LacZ* expression cassette).

The expression constructs contained the sequences below.

The pPGM2 construct contains (5′ TAATAAGAAAAAGATCAC CAATCTTTCTCAGTAAAAAAAGAACAAAAGTTAACATAACAT 3′), pTAR contains the 5′ UTR of the HIV1-TAR gene (5′ GGTTCTCTGGTTAGCCAGATCTGAGCCCGGGAGCTCTCTGGCTAGCTAGGGAACCCACTGCTTAAGCCTCAATAAAGCTTGCCTTGAGTGCTTCAAGTAGTGTGTGCC 3′), 2-hair has a 5′ UTR complex (5′ CTTGGTAAAGGGGGUGGTCTGAGCCCGGGAGCTCTCTGCTGCTTAAGCCTCGGATTTT 3′), pRTN contains the 5′ UTR of the FOAP-11 gene (5′ GGGATTTTTACATCGTCTTGGTAAAGGCGTGTGACCCATAGGTTTTTTAGATCAAACACGTCTTTACAAAGGTGATCTAAGTATCTC 3′), and pBCell contains the 5′ UTR of the BCL-2 gene (5′ GGGGGCCGUGGGGUGGGAGCUGGGGGGGCCGUGGGGU GGGAGCUGGG 3′); the plasmids included an ampicillin resistance gene for selection in DH5α in *E*. *coli* and a *URA3* gene for selection in *S. cerevisiae*.

The yeast growth medium comprised either Synthetic Complete medium with selective amino acids (0.2% amino acid dropout mixture and 0.67% YNB without amino acids) or YP medium (2% peptone and 1% yeast extract). The primary sugar in the media was either 2% galactose or 2% glucose, as indicated. Plasmid extraction for yeast utilized the Omega Biotek^®^ (VWR, Mississauga, ON, Canada) yeast plasmid miniprep kit, while for plasmid extraction in *E. coli*, the Thermofisher^®^ (Mississauga, ON, Canada) and Bio-Basics^®^ (Mississauga, ON, Canada) GeneJET plasmid miniprep kit was used, following the instructions provided by the respective manufacturers.

### 2.3. Western Blotting

Total protein from yeast cells was extracted by using TCA precipitation, as described previously [21,24]. The protein concentration was measured, and abundance was equalized with the BCA protein assay kit (Thermofisher^®^). Then, proteins were separated on 12% SDS-polyacrylamide gel by electrophoresis. The gel was then transferred to a polyvinylidene difluoride membrane (Bio-Rad Laboratories, Hercules, CA, USA) by using a semi-dry transfer apparatus (Bio-Rad). In the next step, 5% non-fat milk in TBS-T (Tris-buffered saline containing 0.1% Tween-20) was used to block the membrane for 1 h at room temperature and was subsequently incubated with primary antibodies overnight at 4 °C. Anti-GFP (Roche, Basel, Switzerland) and anti-actin (Sigma-Aldrich, St. Louis, MO, USA) were used as primary antibodies. After washing with TBS-T, the membrane was incubated with horseradish peroxidase-conjugated secondary antibodies (Santa Cruz Biotechnology, Dallas, TX, USA) for 1 h at room temperature. Protein bands were detected by using an enhanced chemiluminescence reagent (Thermofisher^®^) according to the manufacturer’s instructions. PGK1p was used as a housekeeping protein (internal control). Densitometry analysis was conducted with FUSION FX software (Vilber^®^) (https://www.vilber.com/fusion-fx/, accessed on 22 January 2023). Each experiment was conducted in triplicate, and one-way ANOVA analysis (*p*-value ≤ 0.05) was used to determine significant statistical differences. 

### 2.4. qRT-PCR

Yeast strains were cultured overnight in YPgal with or without LiCl, as performed previously [36]. Total RNA from yeast cells was extracted by using the RNeasy Mini Kit (Qiagen, Hilden, Germany), according to the manufacturer’s instructions. Reverse transcription was performed by using the QuantiTect Reverse Transcription Kit (Qiagen). Real-time quantitative PCR was carried out by using the Power SYBR Green PCR Master Mix (Thermofisher^®^) and the StepOnePlus Real-Time PCR System (Thermofisher^®^). The relative mRNA expression level of each gene was normalized to that of the *PGK1* gene, which served as an internal control. Each experiment was conducted in triplicate, and one-way ANOVA analysis (*p*-value ≤ 0.05) was used to determine significant statistical differences. The qRT-PCR primers used were *PGM2* Forward: GGTGACT CCGTCGCAATTAT; *PGM2* Reverse: CGTCGAACAAAGCACAGAAA. *PGK1* Forward: ATGTCTTTATCTTCAAAGTT; *PGK1* Reverse: TTATTTCTTTTCGGATAAGA.

### 2.5. Drug Sensitivity Analysis

To conduct LiCl sensitivity analysis, specific yeast colonies were cultured for two days at 30 °C in liquid Ypgal medium. After incubation, the liquid cultures were standardized to an OD 0.1 at 600 nm, serially diluted (10^−1^ to 10^−4^), and spot-plated (10 μL per spot) onto Ypgal agar medium with 10 mM LiCl treatment, as performed previously [20,21,37]. The sensitivity to LiCl was assessed by comparing gene deletion strains with the wild-type (WT). The overexpression constructs pCAF20 and pECM32 were transformed into the gene deletion strains to validate the observed sensitivities resulting from the deletion of the candidate genes. For the quantification analysis, colony counting was performed by using 100 μL of cell cultures diluted at a 10^−4^ concentration, which were then spread onto Ypgal plates with and without 10 mM LiCl. After incubation for 48 h at 30 °C, the colonies were counted. Phenethyl isothiocyanate (PEITC) was used as an alternate translation-inhibiting drug by growing yeast cultures overnight in YPD medium. OD standardization and dilutions were performed as they were with LiCl for spot tests and colony counting. Each experiment was conducted in triplicate, and one-way ANOVA analysis (*p*-value ≤ 0.05) was used to determine significant statistical differences.

### 2.6. Quantitative β-Galactosidase Assay

The translation initiation activity of the gene deletion strains was investigated by using *LacZ* reporter constructs, and *LacZ* activity was assessed by using a quantitative *β-galactosidase* assay. The assay was carried out by using O-nitrophenyl-d-galactopyranoside, as performed previously [21,24]. Each experiment was conducted in triplicate, and one-way ANOVA analysis (*p*-value ≤ 0.05) was used to determine significant statistical differences.

### 2.7. Genetic Interaction Analysis

For the analysis of genetic interaction, a 384-formatted template for SGA was utilized. The library of yeast double-deletion mutants was generated as described before [38]. In brief, the query strain MATα, which carried a gene deletion of interest and a clonNAT marker, was mated with ~1000 MATa mutants with non-essential gene deletions, of which ~700 are involved in gene expression pathways, with ~300 being random controls (Supplementary Table S2). Following a series of selection rounds, haploid mutants with double gene deletions were selected. PCR analysis using the pAG25 plasmid as the template was employed to identify successful gene knockout transformations. The Nourseothricin N-acetyl transferase (NAT) and Kanamycin selection markers were used to amplify the clonNAT resistance gene marker. The generated double mutants were grown in sub-inhibitory 3 mM LiCl YPD agar media, a chemical stress condition used for conditional SGA to investigate genes related to LiCl stress conditions.

Phenotypic Suppression Array (PSA) analysis was performed by following the same SGA mating protocol mentioned before but with an additional step of mating an empty plasmid and an overexpression plasmid of a query gene carried by the MATα yeast strain with the MATa single-deletion library. The strains with and without the overexpression plasmid were grown in medium with LiCl treatment to examine whether there was a phenotypic shift with 10 mM LiCl treatment.

### 2.8. Genetic Interaction Data Analysis

The determination of phenotypic changes, such as synthetic illness or synthetic lethality, was performed via normalized colony-size comparisons. The colony-size morphologies were measured by using SGA software [39] (http://sgatools.ccbr.utoronto.ca/, accessed on 22 January 2023), and only size reductions ranging from 30% to 95% were considered. The experiment was repeated in triplicate, with only hits that appeared in at least two replicates being considered. The identified hits were then categorized based on their Gene Ontology molecular function and biological processes, with enrichment being assessed by using Gene Ontology terminology from various tools, including the Genemania database [40] (http://genemania.org, accessed on 26 January 2023) and Gene Ontology Term Finder [30] (https://www.yeastgenome.org/goTermFinder, accessed on 5 February 2023).

## 3. Results

### 3.1. Mutant Strains for CAF20 and ECM32 Are Sensitive to LiCl

Directed chemical genetic methods have been widely used to study novel function(s) for various genes in different cellular processes, including transcription [41], translation [15], and DNA damage repair [42]. By analyzing the sensitivity of gene deletion mutants to a bioactive compound, researchers can identify not only the cellular target pathways and the impact of the compound at the molecular level but also the novel function of genes that are linked to the molecular activity of that compound [20,33,43]. The LiCl sensitivity of yeast mutant strains has been previously used to elucidate novel gene functions associated with the translation of *PGM2* mRNA, which carries a structured 5′ UTR [20,21]. Therefore, to study the activity of the two selected genes, *CAF20* and *ECM32*, in the translation of *PGM2* mRNA, we investigated the sensitivity of yeasts harboring gene deletion mutants for these two genes when challenged with LiCl. 

**Figure 2:**
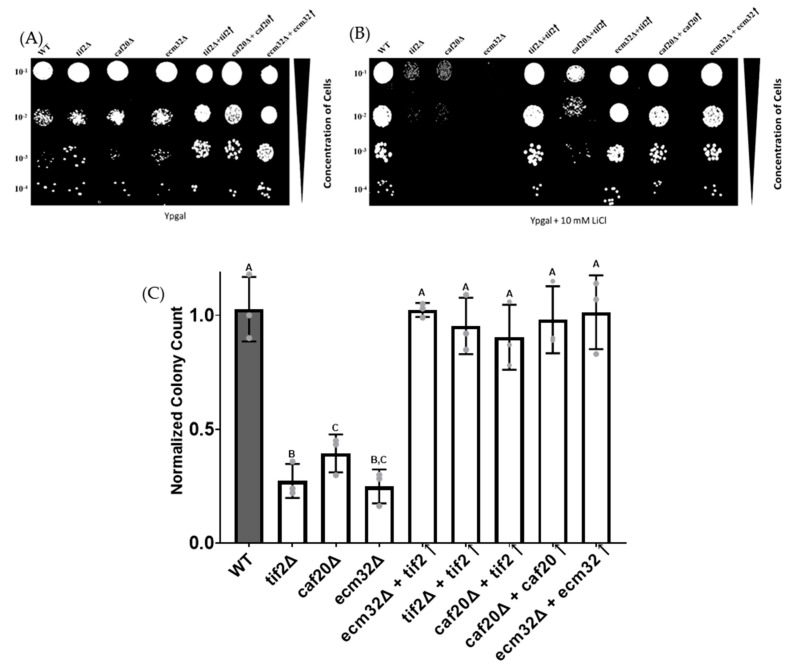
The spot test and colony count analyses show increased sensitivity to LiCl for the *caf20∆* and *ecm32∆* yeast strains. As shown in (**A**), the yeast cells were serially diluted (10^−1^ to 10^−4^) and spotted onto Ypgal media. In (**B**), as in A, but the cells were spotted onto Ypgal medium with 10 mM LiCl. The gene deletion mutants *caf20∆* and *ecm32∆* had reduced growth in the presence of LiCl, with *tif2∆* being used as a control. Phenotypic rescue was observed in mutant strains with overexpressed *TIF2* or when the respective target gene was restored. (**C**) The result of colony count analysis. The deletion mutant strains significantly reduced colony counts in the presence of LiCl compared with the wild-type. Mutant strains with overexpression plasmids had colony counts similar to the wild-type. *p*-Values of *tif2∆* = 4.89 × 10^−5^, *caf20∆ =* 7.53 × 10^−5^, and *ecm32∆* = 2.65 × 10^−5^ were obtained, with all other results being insignificant. Letters above the bars indicate statistical significance. Bars that share the same letter do not have a significant difference between them, while bars with different letters have a significant difference between them. The experiments were performed in triplicate, and the data are presented as the mean values. Bars represent the standard deviations (SDs). Statistical analysis was performed by using one-way analysis of variance (ANOVA), with multiple comparisons followed by Tukey’s post hoc test. A *p*-value < 0.05 was considered to indicate statistical significance.

The spot test analysis revealed that the deletion of *CAF20* and *ECM32* resulted in a significant reduction in cell growth in galactose-containing media supplemented with 10 mM LiCl (Figure 2A,B), indicating increased cell sensitivity to LiCl. *TIF2* is a deletion mutant used as a positive control. *TIF2* encodes *eIF4A*, an RNA helicase that is required for the translation of structured mRNA [44]. It is possible that the observed phenotype was caused by unintentional secondary mutations. To address this, we performed a rescue experiment to further study the association between LiCl sensitivity and the target gene deletions by reintroducing the deleted genes back into the cells. This was performed by using overexpression plasmids for the target genes. Our results demonstrate that the reintroduction of the deleted genes compensated for the previously impaired growth defects, providing further evidence of the functional relationship between these genes and LiCl sensitivity (Figure 2A,B). The analysis of colony count measurements further support these findings by providing a quantitative viewpoint (Figure 2C).

Previous studies have demonstrated that the overexpression of *TIF2* can reverse the sensitivity of yeast deletion strains for different genes thought to participate in the translation of *PGM2* and other structured mRNAs in LiCl [44]. Next, we investigated whether the overexpression of *TIF2* can compensate for the LiCl-sensitive phenotypes associated with the *CAF20* and *ECM32* mutant strains (Figure 2A,B). The introduction of a plasmid carrying the *TIF2* gene restored the fitness of the deletion mutant strains, indicating that the overexpression of *TIF2* can compensate for the deletion of *CAF20* or *ECM32*. The analysis of colony count measurements further confirmed these results (Figure 2C).

LiCl sensitivity in *S. cerevisiae* is suggested to be caused by reduced activity and expression of *PGM2*—a gene which encodes an enzyme that converts glucose-1-phosphate into glucose-6-phosphate, which is critical for galactose metabolism [45]. Reduced *PGM2* activity results in the accumulation of toxic intermediates when the primary carbon source is galactose. To investigate galactose-dependent cell sensitivity, we also examined the response of yeast mutant strains to LiCl when the carbon source is glucose. We observed no significant growth change for mutant strains compared with the control strain (Supplemental Figure S1), indicating that the sensitive phenotype for the mutants is galactose-dependent.

### 3.2. Deletion Mutants for CAF20 and ECM32 Are Sensitive to Phenethyl Isothiocyanate 

**Figure 3:**
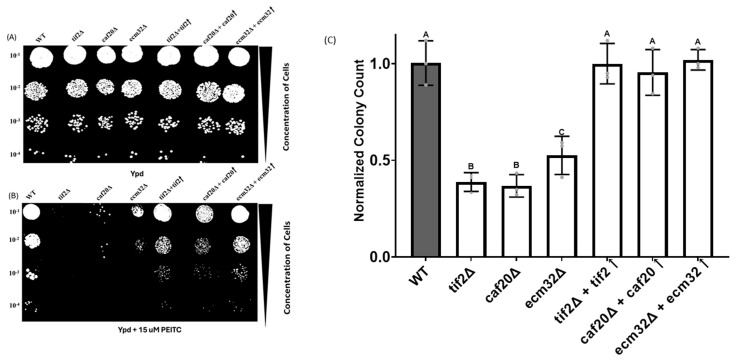
The spot test and colony count analysis showed increased sensitivity to PEITC for the *tif2∆*, *caf20∆*, and *ecm32∆* yeast strains compared with the wild-type. (**A**,**B**) Serially diluted yeast cells (10^−1^ to 10^−4^) and cells spotted onto YPD media and YPD media with 15 μM PEITC, respectively. The gene deletion mutants *caf20∆* and *ecm32∆* had reduced growth in the presence of PEITC, in (**B**) when compared with PEITC-free conditions. *tif2∆* was used as a positive control. Partial phenotypic rescue was observed in the mutant strains when the respective target gene was restored by using a plasmid. (**C**) Colony count analysis in PEITC growth medium. Mutant deletion strains had a significantly reduced number of colonies in the presence of PEITC compared with the wild-type. Mutant strains with reintroduced genes had colony counts similar to the wild-type. *p*-Values of *tif2∆ =* 1.86 × 10^−7^, *caf20∆* = 6.41 × 10^−6^, and *ecm32∆* = 9.74 × 10^−6^ were obtained, with all other results being insignificant. Letters above the bars indicate statistical significance. Bars that share the same letter do not have a significant difference between them, while bars with different letters have a significant difference between them. All experiments were performed in triplicate, and the data are presented as the mean values, with bars representing the standard deviations (SDs). Statistical analysis was performed by using one-way analysis of variance (ANOVA), with multiple comparisons followed by Tukey’s post hoc test. A *p*-value < 0.05 was considered to indicate statistical significance.

PEITC is a naturally occurring compound found in cruciferous vegetables. It is reported to preferentially inhibit the cap-dependent translation of certain mRNAs that carry complex secondary structures, such as *MYC* [46,47]. Deletion mutant strains for genes involved in the translation of *PGM2* and several other structured 5′ UTRs have shown increased sensitivity to PEITC [24]. Therefore, we next evaluated the *CAF20*- and *ECM32*-deletion mutants for their sensitivity to PEITC. As indicated in Figure 3A,B, both *caf20∆* and *ecm32∆* displayed increased sensitivity to 15 μM PEITC. As mentioned above, the deletion of *TIF2* was used as a control. Additionally, the observed sensitivity induced by PEITC treatment was reversed when the deleted genes were reintroduced into their respective mutant strains (Figure 3A,B), further validating that the observed phenotypes are linked to the deleted genes and do not represent a consequence of other unintended genomic alterations.

### 3.3. CAF20 and ECM32 Regulate Translation of PGM2 mRNA

**Figure 4:**
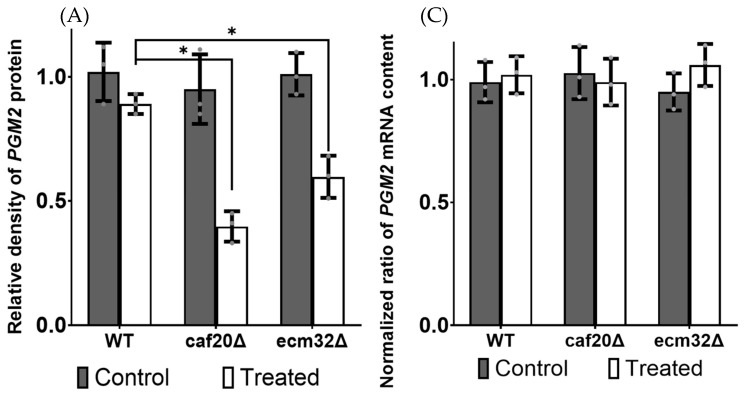

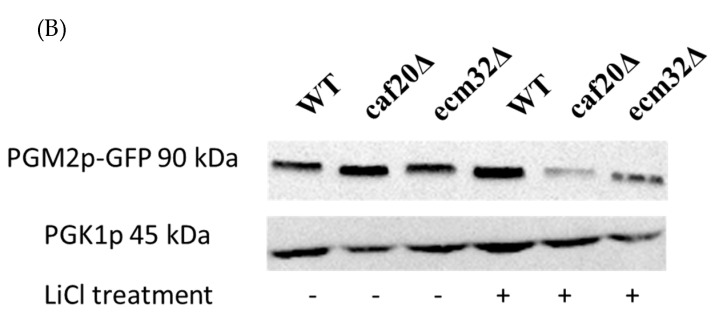
*PGM2* protein and mRNA content analyses based on Western blot and qRT-PCR with and without 10 mM LiCl treatment. (**A**) The *PGM2*-GFP protein level was determined via Western blot analysis, shown in (**B**). The deletion mutants *caf20∆* and *ecm32∆* had significantly reduced *PGM2*-GFP levels when treated with 10 mM LiCl compared with the wild-type. *PGK1*, a commonly used housekeeping protein, is the internal control, with results normalized to it. The *p*-values for strains treated with LiCl were *caf20∆* = 2.59 × 10^−6^ and *ecm32∆* = 7.83 × 10^−5^, with the WT being insignificant. As shown in (**C**), qRT-PCR determined the *PGM2*-*GFP* mRNA levels. No significant differences in normalized *PGM2* mRNA contents were found in the yeast strains. (**C**) The QRT-PCR results are insignificant. All experiments were performed in triplicate, and the data are presented as the mean values, with bars representing the standard deviations (SDs). Asterix (*) represent significant difference between the bars. Values are related to that of the wild-type. Statistical analysis was performed by using one-way analysis of variance (ANOVA), with multiple comparisons followed by Tukey’s post hoc test. A *p*-value < 0.05 was considered to indicate statistical significance.

*PGM2* mRNA is predicted to carry a structure in its 5′ UTR [20,24]. Consequently, the deletion of genes involved in the translation of structured mRNAs has been shown to contribute to a reduction in the protein content of *PGM2* in the cells that are challenged by LiCl [20,21,24,36]. To assess the potential impact of *CAF20* and *ECM32* deletion on *PGM2* expression, we employed Western blot analysis. We first measured the protein levels of GFP-tagged *PGM2* under native conditions and after exposure to LiCl. The results shown in Figure 4A indicate that the deletion of the *CAF20* and *ECM32* genes had no significant effect on *PGM2* expression under standard laboratory growth conditions. However, when LiCl was added, the deletion of *CAF20* or *ECM32* reduced the protein levels for *PGM2* by approximately 50%. Since the observed difference may be explained by mRNA content levels, we also evaluated the mRNA content for *PGM2* mRNA. This was performed by using qRT-PCR analysis. No significant alterations in the amount of *PGM2* mRNA content were observed (Figure 4B). These observations are consistent with previous studies that demonstrated the inhibition of *PGM2* 5′ UTR reporter expression at the translational level when genes associated with the translation of structured mRNAs were deleted [20,21,24,36]. 

### 3.4. Effect of CAF20 and ECM32 Deletion on Translation of Reporter mRNAs with Structured 5′ UTRs 

**Figure 5:**
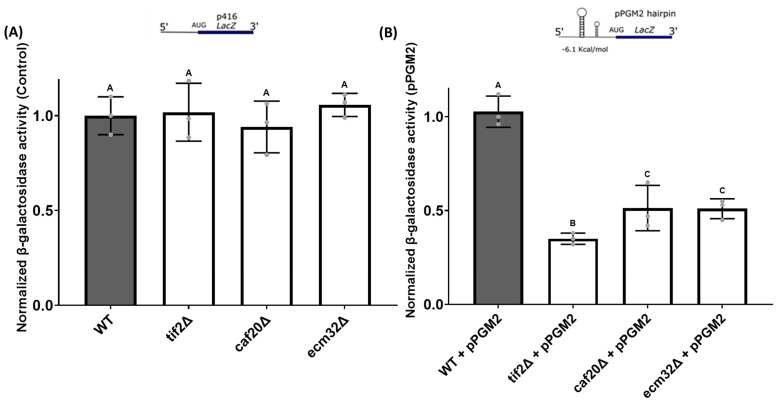
Quantitative *β-galactosidase* activity of yeast strains carrying an expression plasmid with and without a 5′ UTR *PGM2* hairpin structure. As shown in (**A**), there was no significant difference in *β-galactosidase* activity between the yeast strains carrying the control *LacZ* cassette without a structured 5′ UTR. The differences between the results are insignificant. As shown in (**B**), there were significant differences in *β-galactosidase* activity for the mutant strains *tif2∆*, *caf20∆*, and *ecm32∆*, compared with the wild-type when *LacZ* is under the translational control of a *PGM2* 5′ UTR. *p*-Values of *tif2∆* + pPGM2 *=* 9.49 × 10^−6^, *caf20∆* + pPGM2 = 4.29 × 10^−5^, and *ecm32∆* + pPGM2 = 4.13 × 10^−5^ compared with the WT were obtained. Letters above the bars indicate statistical significance. Bars that share the same letter do not have a significant difference between them, while bars with different letters have a significant difference between them. All experiments were performed in triplicate, and the data are presented as the mean values, with bars representing the standard deviations (SDs). Values are normalized to that of the wild-type. Statistical analysis was performed by using one-way analysis of variance (ANOVA), with multiple comparisons followed by Tukey’s post hoc test. A *p*-value < 0.05 was considered to indicate statistical significance.

The 5′ UTR of *PGM2* mRNA is predicted to contain a stem-loop structure [48]. The expression of this mRNA was found to be significantly reduced upon the deletion of *TIF2*, a helicase protein responsible for unwinding mRNA structures during translation initiation. Also, it is reported that a number of genes that regulate the translation of *PGM2* mRNA exert their activities through the 5′ UTR of its mRNA [20,21,24,36]. Therefore, to further study the effect of *CAF20* and *ECM32* on the regulation of *PGM2* mRNA translation, we employed a *LacZ* expression reporter system that is under the translational control of the *PGM2* 5′ UTR. This expression plasmid and a control plasmid without a structured 5′ UTR were transformed into the target yeast mutant and the wild-type strains. *LacZ* gene expression was measured by using a quantitative *β-galactosidase* assay. No significant variation in translation activity for the control plasmid was observed (Figure 5A). However, a significant reduction in *β-galactosidase* activity for the *caf20∆* and *ecm32∆* strains, similar to the *tif2∆* control, was observed with the expression plasmid carrying *LacZ* under the translation control of the *PGM2* 5′ UTR (Figure 5B). 

**Figure 6:**
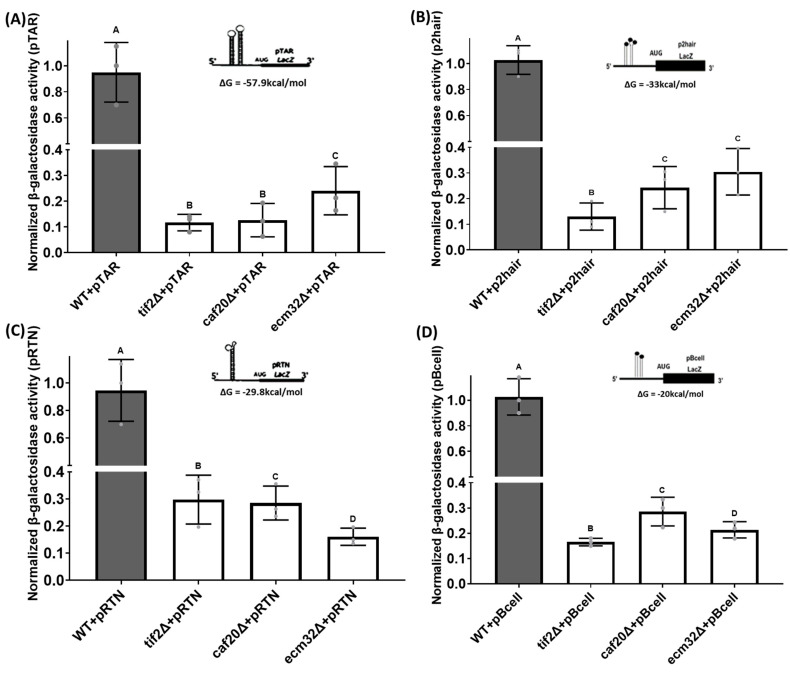
The quantitative *β-galactosidase* activity of yeast strains carrying an expression plasmid with different structured 5′ UTRs upstream of the *LacZ* reporter. (**A**) All mutant yeast strains that carry a pTAR containing a complex 5′ UTR structure from *HIV1-TAR* had reduced *β-galactosidase* activity compared with the wild-type. *p*-Values of *tif2∆* + pTAR *=* 8.23 × 10^−7^, *caf20∆* + pTAR = 3.91 × 10^−6^, and *ecm32∆* + pTAR = 6.30 × 10^−5^ were obtained. (**B**) All mutant yeast strains that carry a p2hair containing a synthetic complex 5′ UTR structure 2hair had reduced *β-galactosidase* activity compared with the wild-type. *p*-Values of *tif2∆* + p2hair *=* 1.83 × 10^−7^, *caf20∆* + p2hair = 9.59 × 10^−6^, and *ecm32∆* + p2hair = 2.72 × 10^−5^ were obtained. (**C**) All mutant yeast strains that carry a pRTN containing a complex 5′ UTR structure from *FOAP-11* had reduced *β-galactosidase* activity compared with the wild-type. *p*-Values of *tif2∆* + pRTN *=* 2.45 × 10^−5^, *caf20∆* + pRTN = 3.96 × 10^−5^, and *ecm32∆* + pRTN = 5.23 × 10^−6^ were obtained. (**D**) All mutant yeast strains that carry a pBcell containing a complex 5′ UTR structure from *BCL-2* had reduced *β-galactosidase* activity compared with the wild-type. *p*-Values of *tif2∆* + pBcell *=* 3.13 × 10^−7^, *caf20∆* + pBcell = 4.55 × 10^−5^, and *ecm32∆* + pBcell = 1.90 × 10^−5^ were obtained. Letters above the bars indicate statistical significance. Bars that share the same letter do not have a significant difference between them, while bars with different letters have a significant difference between them. All experiments were performed in triplicate, and the data are presented as the mean values, with bars representing the standard deviations (SDs). Statistical analysis was performed by using one-way analysis of variance (ANOVA), with multiple comparisons followed by Tukey’s post hoc test. A *p*-value < 0.05 was considered to indicate statistical significance.

To explore the impact of *CAF20* and *ECM32* on the translation of other structured mRNAs, we examined four additional constructs, each featuring a distinct structure at their 5′ UTR. These constructs included pTAR, which possesses a structure originating from the *HIV1-TAR* mRNA 5′ UTR, with a ΔG = −57.9 kcal/mol; p2hair, a synthetically designed structure with high complexity and a ΔG = −33 kcal/mol; pRTN, containing the structured 5′ UTR of *RTN4IP1* mRNA, with a ΔG = −29.8 kcal/mol; and pBcell, which contains a structure originating from *BCL*-*2* mRNA, with a ΔG = −20 kcal/mol. Our analysis showed that the deletion of *CAF20* and *ECM32* significantly decreased the expression of the reporter genes with a complex 5′ UTR for all four structured mRNAs (Figure 6A–D). As performed previously, the deletion of *TIF2* was used as a positive control. Of interest, the reduction in activities for different mutants seemed to be different for certain structures. For example, expressions derived from pTAR and pRTN were approximately 10% and 30% for the deletion of *CAF20* and 30% and 15%, respectively, for the deletion of *ECM32.*

### 3.5. Genetic Interaction Analysis Further Links Additional Functions of CAF20 and ECM32 to Pathways Related to Translation

Genetic interaction (GI) analysis relies on the idea that cells gain resilience and adaptability to spontaneous detrimental mutations based on the presence of parallel pathways, thereby maintaining cellular homeostasis and viability [49,50]. This principle dictates that when one gene in a pathway compensates for the absence of another gene in a separate pathway, the mutant cell can survive. When cell fitness decreases (sickness) or cell death (lethality) is observed from deleting two genes that individually seem to have no fitness consequence, it is generally thought that those genes function on parallel compensatory pathways [51,52]. This type of interaction is known as negative genetic interaction (nGI) [53]. nGIs are frequently used to investigate gene function and pathway interactions [3,54].

To analyze nGIs in yeast, a high-throughput analysis was conducted by crossing two yeast mating types, MATα and MATa. The target gene deletion is carried by MATα and is crossed with various single-deletion mutants of MATa to produce double-deletion mutants. Strain fitness is measured by colony size [55]. This method investigates the genetic connections between *CAF20* and *ECM32* and 1000 other genes, respectively, including 700 genes connected to the gene expression pathway and a random group of 300 genes for control (Supplementary Table S2).

**Figure 7:**
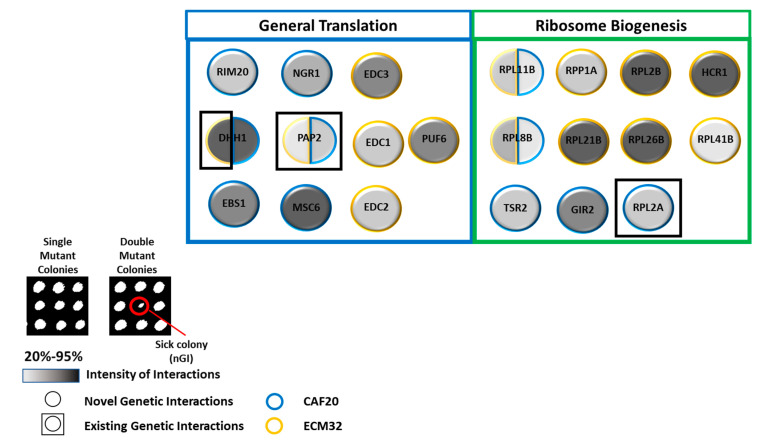
Negative genetic interactions for *CAF20* and *ECM32.* A collection of interactors falls in the categories of general translation and ribosome biogenesis for *CAF20* (*p* = 3.83 × 10^−7^) and *ECM32* (*p* = 4.52 × 10^−7^). *DHH1*, *PAP2*, *RPL8B*, and *RPL11B* are mutual hits shared between *CAF20* and *ECM32*. Circles represent genes, while nGIs between *CAF20* or *ECM32* are represented by the circle’s perimeter coloration: blue for *CAF20* and yellow for *ECM32*. Existing (previously published) interactions are differentiated by a black box outlining the circles. The strength of the interaction is reflected by the gradient, with darker coloration indicating a higher level of decreased fitness. A representative nGI is indicated in the inset by the red circle.

Our analysis identified several interesting nGIs and common gene hits between *CAF20* and *ECM32* (Figure 7). The functional enrichment analysis of the hits revealed that many of them are linked to general translation and ribosome biogenesis processes.

For *CAF20*, we identified nGIs with genes *EBS1* and *NGR1*, among others (Figure 7). *EBS1* is involved in translation initiation and nonsense-mediated decay [56]. *NGR1* encodes an RNA-binding protein that interacts with the *DHH1* helicase protein, which has been implicated in *POR1* mRNA decay [57].

*ECM32* interacted with *EDC1*, -*2*, and -*3*; *HCR1*; and a series of other genes (Figure 7). The *EDC* family of proteins are RNA-binding proteins involved in the decapping of mRNA. *EDC1* and *EDC2* are paralogues and enhance the activity of *DCP1*p and *DCP2*p decapping enzymes [58]. *EDC3* also enhances *DCP1* decapping activity and mediates *RPS28B* mRNA decay [59]. *HCR1* encodes a component of *eIF3* involved in 20S pre-rRNA processing [60].

A number of common interactors between *CAF20* and *ECM32* were found, including *DHH1*, *PAP2*, and RPL11B (Figure 7). *DHH1* encodes a DEAD-box helicase involved in translation regulation [61]. *PAP2* encodes a non-canonical poly(A) polymerase that contributes to 3′–5′ RNA helicase activity [62]. A ribosomal 60S subunit, *RPL11B*, is involved in ribosomal assembly and translation regulation [63].

**Figure 8:**
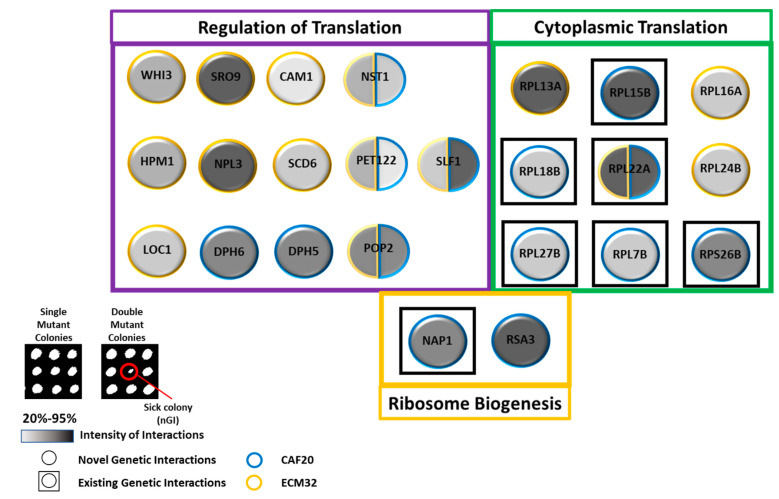
Conditional negative genetic interactions for *CAF20* and *ECM32* under sub-inhibitory LiCl concentration of 3 mM. A collection of interactors falls in the categories of regulation of translation for *CAF20* (*p* = 7.89 × 10^−7^) and *ECM32* (*p* = 1.20 × 10^−8^); cytoplasmic translation for *CAF20* (*p* = 1.41 × 10^−7^) and *ECM32* (*p* = 5.67 × 10^−7^); ribosome biogenesis for *CAF20* (*p* = 4.93 × 10^−7^). *NST1*, *PET122*, *POP2*, *RPL22A*, and *SLF1* are mutual hits shared between *CAF20* and *ECM32*. Circles represent genes, nGIs for *CAF20* or *ECM32* are represented by the circle’s perimeter coloration: blue for *CAF20* and yellow for *ECM32*. Existing (previously published) interactions are differentiated by a black box outlining the circles. The strength of the interaction is reflected by the gradient, with darker coloration indicating decreased fitness. A representative nGI is indicated in the inset by the red circle.

Conditional nGIs are triggered by meeting a condition that allows for a functional interaction, for instance, an environmental stressor [35,64]. Conditions can include factors such as lack of nutrients, acidity, exposure to extreme temperatures, or the presence of bioactive chemicals. These conditions help to establish functional links between genes that are formed only in response to specific conditions. For example, the activity of a number of genes is known to change in response to DNA damage or oxidative stress [54,65]. In many cases, environmental changes can cause non-essential genes to become vital for cell survival under that specific condition. Such condition-dependent functional relationships form the basis of conditional nGIs. In our analysis, we assessed nGIs by using a sub-inhibitory concentration of LiCl (3 mM). New nGIs formed in response to LiCl are shown in Figure 8. 

The sub-inhibitory concentration of LiCl revealed a new set of nGIs for *CAF20* with several translation-related genes, including *RSA3* and *RPS26B* (Figure 8). *RSA3* encodes a protein that binds to helicase *DBP6*p, which plays a role in ribosome biogenesis [66]. *RPS26B* encodes a ribosomal protein associated with the 40S ribosomal subunit [67]. A new group of interactors that played a role in the regulation of translation were also identified, such as *DPH5* and *DPH6* (Figure 8). *DPH5* and *DPH6* are important genes required for Dipthamide biosynthesis for *eIF2* [68,69].

*ECM32* also had a series of new nGIs with several translation genes: *CAM1* and *SRO9*, along with other genes (Figure 8). *CAM1* is the gamma subunit of *eEF1B* [70]. *SRO9* is associated with translating ribosomes and functions as a cytoplasmic RNA-binding protein [71]. The interactors of *ECM32* involved in translation regulation were also revealed by conditional negative interactors such as *NPL3*, *SCD6*, and others (Figure 8). *NPL3* is involved in translation initiation inhibition by binding to *eIF4G*, promotes elongation, and regulates termination [72]. The gene product of *SCD6* binds to *eIF4G* to inhibit preinitiation complex formation during translation initiation [73].

Common interactors between *CAF20* and *ECM32* were also found, such as *NST1* and *SLF1*, among others (Figure 8). *NST1* encodes a translational inhibitor [74]. The *SLF1* gene product binds to RNA associated with polysomes and is thought to be involved in the regulation of translation [75].

**Figure 9:**
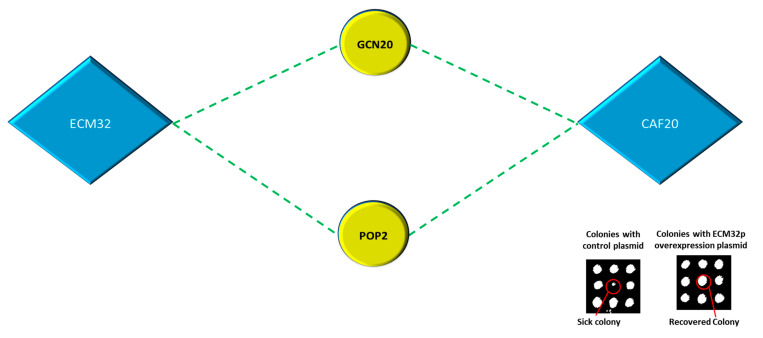
The overexpression of *CAF20* and *ECM32* reverses cell sensitivity induced by 10 mM LiCl for *GCN20* and *POP2*. Both *GCN20* and *POP2* have functions associated with the regulation of translation. The red circle in the inset is a representative interaction; it depicts the phenotype regained after the introduction of an *ECM32* overexpression plasmid in the presence of 10 mM LiCl.

The Phenotypic Suppression Array (PSA) analysis investigates a distinct type of interaction in which the overexpression of a particular gene makes up for the absence of another gene and compensates for an observed phenotype [12]. This type of interaction can be very informative, as it can reveal a certain functional overlap between two genes. In this study, we exposed the mutant gene array to 10 mM LiCl and found that several mutant strains displayed increased cell sensitivity. To counteract this sensitive phenotype, we introduced overexpression plasmids for *CAF20* or *ECM32*. The introduction of *CAF20* or *ECM32* overexpression plasmids successfully restored the sensitivity caused by LiCl treatment for two gene deletion mutants, namely, *GCN20* and *POP2* (Figure 9). *GCN20* encodes a positive regulator of *GCN2*p kinase that phosphorylates the alpha subunit of *eIF2* in response to stress and promotes the translation of specific genes while restricting global translation initiation [76]. *POP2* is a subunit of the *CCR4*-Not complex involved in mRNA deadenylation, along with various other activities [77].

## 4. Discussion

The regulation of translation via structured mRNA is multifaceted, multiscale, and influenced by 5′ UTR secondary structures that impede translation initiation. In this study, we demonstrated that *CAF20* and *ECM32* influence the translation of *PGM2* mRNA and several other synthetic structured 5′ UTRs. These findings are consistent with previous studies demonstrating the impact of RNA helicases and other translation factors on the efficiency of structured *PGM2* mRNA translation and of several synthetic constructs with complex 5′ UTR structures [20,21,24]. From the interpretation of this result, some mechanisms could be suggested concerning how *CAF20* and *ECM32* regulate the translation of structured mRNAs. One possibility is that *CAF20* and *ECM32* interact with components of the translation initiation machinery and may function as helicase modulators that affect the unwinding of several different 5′ UTR structures. This is consistent with previous studies investigating translation regulators affecting helicase activity. Another possibility is that *CAF20* and *ECM32* could alter ribosome dynamics, making the translation of structured mRNAs less efficient in their absence. In line with this hypothesis, we found that *CAF20* and *ECM32* have interactions with various ribosomal proteins, suggesting that these genes functionally relate to the ribosome’s ability to process the structured mRNAs used in this study. We found that the overexpression of *TIF2* was able to restore the fitness of *CAF20-* and *ECM32*-deletion strains in LiCl exposure. As *TIF2* was able to compensate for the loss of these factors, it suggests that *CAF20* and *ECM32* may act as modulators of translation initiation, suggesting they may have regulatory roles in the translation complex. *CAF20* and *ECM32* may have functional redundancy with respect to *TIF2*. It is possible that *CAF20* and *ECM32* enhance the activity of *TIF2*, resulting in similar phenotypes when *TIF2* is overexpressed or when *CAF20* or *ECM32* are present. Further studies can explore whether these proteins physically interact or affect overlapping subsets of 5′ UTR mRNA structures. Interestingly, the deletion of *CAF20* and *ECM32* did not uniformly affect the translation of each of the tested mRNA constructs, each of which has diverse secondary structures in the 5′ UTR. This could be due to differences in the properties of the secondary structures, for example, free energy values, loop configurations, and GC content. Structural diversity within 5′ UTRs can, therefore, confer a differential sensitivity toward the loss of these regulatory genes. The diverse effects observed from the range of different structured 5′ UTRs suggest that regulatory networks that influence translation are much more complex than previously thought. The overexpression of *CAF20* and *ECM32* reverses the LiCl sensitivity induced by *gcn20Δ* and *pop2Δ*, suggesting that these genes may functionally interact in some capacity. One possibility is that these proteins may have some level of functional redundancies; hence, the overexpression of one may compensate for the absence of the other. One possibility is that the mRNA and/or protein content levels for *CAF20* and *ECM32* might be altered in *gcn20Δ* and *pop2Δ* mutant strains. Future endeavors can explore the functional relationship between these genes perhaps by investigating whether *gcn20Δ* and *pop2Δ* mutations alter the mRNA or protein expression of *CAF20* and *ECM32*, providing further insights into the molecular basis of their interactions. Alongside our structured mRNA constructs, exploring complex mRNA hairpins of yeast orthologues or functionally equivalent genes such as human *BCL2* would be of interest. Future studies may determine whether *CAF20* and *ECM32* the influence translation efficiency of these orthologues; however, this remains speculative. Moreover, the hypersensitivity of *CAF20* and *ECM32* mutants to LiCl and PEITC seems to highlight the role of other environmental and chemical stressors possibly involved in modulating the translation of structured mRNAs. While insights into the regulation of some structured mRNAs is provided in this study, it is limited by the use of yeast as the model for these processes. For the translational relevance of these findings to human cells, this topic would require further investigation, using mammalian systems to validate the roles of *CAF20* and *ECM32* homologs in humans. Future studies could investigate how these regulatory mechanisms extend into human cells and whether homologs of *CAF20* and *ECM32* play similar roles in the translation of structured mRNAs in disease contexts. Targeting factors similar to *CAF20* and *ECM32* may offer new strategies for enhancing translation efficiency or mRNA used in vaccines or gene therapies in clinical environments. Additionally, as LiCl is a commonly used therapeutic in bipolar disorder, the human homologs of *CAF20* and *ECM32* may play a role in how lithium exerts its effects on neuronal function. Further studies on these homologs may provide further insights into lithium’s therapeutic mechanisms, potentially revealing novel targets of drug development for mental ailments such as bipolar disorder. Another direction might be investigating other chemical compounds that affect the translation of structured mRNAs for further insights into therapeutic strategies, in particular for conditions like bipolar disorder, where LiCl already plays a therapeutic role. The elucidation of how structured mRNA translation is regulated under various conditions of stress may reveal novel targets for drug development.

## 5. Conclusions

Altogether, the GIs observed for *CAF20* or *ECM32* suggest a functional relationship between these genes and a series of other genes involved in translation. Under stress conditions caused by a sub-inhibitory concentration of LiCl, a new group of interactors involved in the regulation of the translation of *PGM2* mRNA and the unwinding of different 5′ UTRs used in this study is uncovered for both genes, suggesting that their activities have some role in the regulation of translation. 

## Data Availability

All data generated and/or analyzed during this study are included in this research article and/or its Supplementary Materials Files.

## Supplementary Materials

The following supporting information can be downloaded at: https://www.mdpi.com/article/10.3390/biology13110884/s1, Figure S1. Mutant strain LiCl sensitivity analyses in YPD media. LiCl sensitivity is not observed for deletion strains of CAF20 and ECM32 in media with glucose as the primary carbon source compared to WT and TIF2 mutant. Spot analysis is conducted with at least three replicates (n ≥ 3) with similar results. Table S1. Input list of genes involved in translation used in computational screening. Table S2. List of mutant strains in gene expression and random arrays.
