# Supplementary material for: Investigating the Activities of CAF20 and ECM32 in the Regulation of PGM2 mRNA Translation
Source: Biology (Basel). 2024 Oct 30;13(11):884. doi: 10.3390/biology13110884 (PMC11592143; doi:10.3390/biology13110884)
Supplement: Supplementary file 1 [file biology-13-00884-s001.zip › biology-3243057-supplementary.pdf]

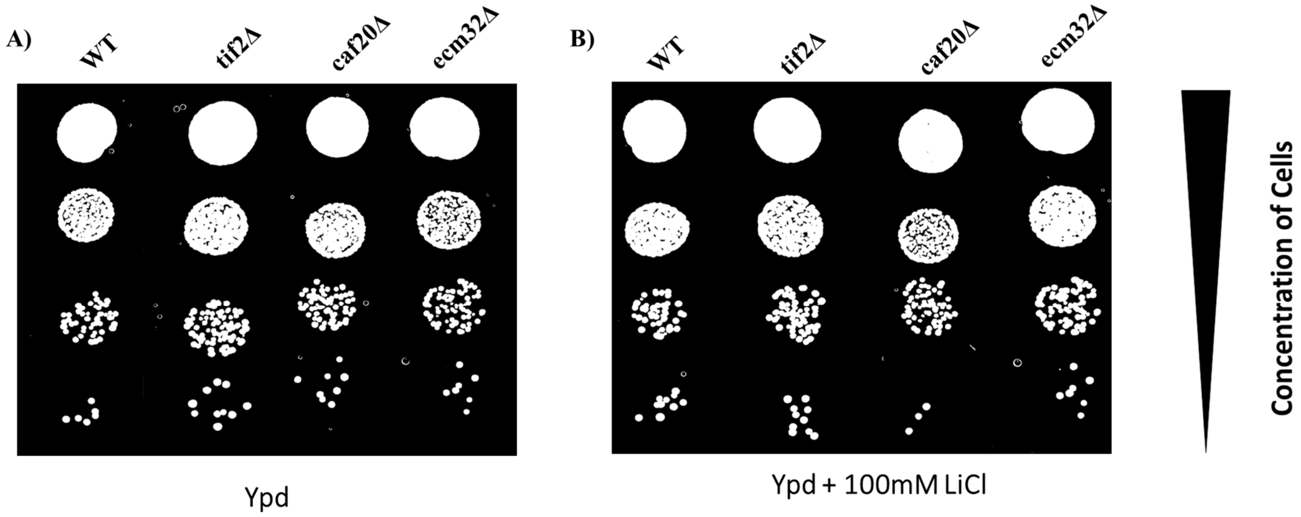

Supplementary Figure S1. Mutant strain LiCl sensitivity analyses in YPD media. LiCl sensitivity is not observed for deletion strains of *CAF20* and *ECM32* in media with glucose as the primary carbon source compared to WT and *TIF2* mutant. Spot analysis is conducted with at least three replicates (n ≥3) with similar results.

Table S1. Input list of genes involved in translation used in computational screening.

YDL160C	YNR038W
YDR332W	YER172C
YDR291W	YER013W
YDR174W	YBR142W
YER164W	YDL031W
YGL078C	YLL008W
YGL101W	YDL084W
YFR038W	YMR128W
YGL251C	YDR243C
YHR031C	YDR021W
YJL138C	YGL171W
YIR002C	YPL029W
YKR059W	YJL050W
YKR024C	YGL120C
YKL017C	YHR065C
YLR221C	YHR169W
YLR247C	YOR046C
YLR032W	YNL112W
YLR398C	YBR237W
YMR080C	YNR011C
YMR190C	YLR419W
YNL273W	YOR204W
YNL224C	YKR086W
YNL218W	YOL147C
YNL339C	YOR275C
YOL095C	YGL063W
YPL119C	YDR471W
YPL008W	YPL074W
YGR271W	YPR096C
YJL092W	YBL016W

**YJL033W**	**YPL203W**
**YKL078W**	**YKR086W**
**YMR290C**	**YFR001W**
**YGL064C**	**YDR496C**
**YLR276C**	**YMR128W**
**YFL002C**	

Table S2. List of mutant strains in gene expression and random arrays.

Genes found in the gene expression array		Genes found in the random array	
YLR085C	YER069W	YJL130C	YMR105C
YER088C	YDR496C	YAL005C	YMR105C
YPL086C	YEL066W	YAL017W	YMR105C
YFL031W	YER117W	YAL036C	YMR119W-A
YMR172W	YER027C	YBL013W	YMR120C
YOL012C	YGL105W	YBL066C	YMR121C
YLR451W	YGL236C	YBR025C	YMR122C
YGL035C	YGR085C	YBR031W	YMR123W
YGL151W	YGR084C	YBR061C	YMR124W
YGL013C	YGL115W	YBR101C	YMR126C
YJL089W	YGR118W	YBR121C	YMR127C
YMR179W	YGR034W	YBR146W	YMR129W
YOR047C	YGR027C	YBR181C	YMR130W
YGL162W	YGL196W	YBR187W	YMR132C
YAL059W	YGL195W	YBR212W	YMR194W
YKR059W	YHR189W	YBR294W	YMR207C
YJL191W	YHR075C	YBR295W	YMR209C
YLR287C-A	YGR201C	YBR296C	YMR210W
YNL162W	YHL039W	YBR297W	YMR214W
YOL045W	YHL033C	YBR301W	YMR215W
YFL034C-A	YIL038C	YCL001W	YMR216C
YNL069C	YIL070C	YCL001W-A	YMR219W
YML068W	YBR118W	YCL002C	YMR221C
YBL032W	YDR477W	YCL006C	YMR222C
YGL248W	YIL064W	YCL028W	YMR223W
YJL190C	YJL136C	YCL029C	YMR224C
YMR012W	YJR014W	YCL037C	YMR256C
YNL299W	YIR038C	YCR003W	YMR258C
YFR032C-A	YJL162C	YCR026C	YMR259C
YDR447C	YJR051W	YCR027C	YMR261C
YDL219W	YJR094W-A	YCR028C-A	YMR262W
YNL277W	YIR001C	YCR032W	YMR263W
YDR378C	YJL073W	YCR033W	YMR264W
YLR172C	YLR012C	YCR034W	YMR265C
YPL090C	YKL185W	YCR036W	YMR266W
YDR450W	YKL202W	YCR037C	YMR269W
YMR116C	YKL201C	YCR046C	YMR271C
YER081W	YKL198C	YDL083C	YNL037C
YCR003W	YJR129C	YDL110C	YNL040W
YER056C-A	YKR091W	YDL134C	YNL041C
YEL050C	YKR087C	YDL185W	YNL043C
YKL074C	YKR084C	YDL188C	YNL044W

YLR333C	YKR101W	YDL191W	YNL045W
YOR185C	YKL006W	YDL224C	YNL046W
YOL080C	YKL156W	YDR025W	YNL049C
YDR494W	YKL056C	YDR152W	YNL050C
YFR009W	YGR271W	YDR179W-A	YNL051W
YNR022C	YNL014W	YDR234W	YNL052W
YJR145C	YLR327C	YDR239C	YNL096C
YPR043W	YLR264W	YDR241W	YNL284C
YLR192C	YLR362W	YDR244W	YNR041C
YLR303W	YLR281C	YDR245W	YNR052C
YAR018C	YLR388W	YDR247W	YOR158W
YHR077C	YLR185W	YDR248C	YOR197W
YGL232W	YLR425W	YDR249C	YOR202W
YKL081W	YLR441C	YDR250C	YOR202W
YLR335W	YMR080C	YDR251W	YOR202W
YMR302C	YMR230W	YDR252W	YOR202W
YPL048W	YMR226C	YDR275W	YOR208W
YPL079W	YMR225C	YDR282C	YOR209C
YER131W	YMR020W	YDR293C	YOR212W
YBR084C-A	YNL288W	YDR357C	YOR213C
YKL204W	YMR269W	YDR358W	YOR214C
YGR178C	YNL040W	YDR359C	YOR215C
YJR150C	YNL067W	YDR360W	YOR216C
WT	YNL063W	YDR363W	YOR219C
YLL045C	YNL304W	YDR363W-A	YOR221C
YLR344W	YNL223W	YDR368W	YOR313C
YOR293W	YNL339C	YDR369C	YOR314W
YBR130C	YNL104C	YDR370C	YOR315W
YML063W	YML129C	YDR371W	YOR316C
YIL052C	YNL096C	YDR372C	YOR317W
YCR031C	YNL239W	YDR400W	YOR318C
YLR406C	YOR091W	YDR422C	YOR320C
YLR039C	YNR020C	YDR432W	YOR321W
YDR515W	YOR107W	YDR450W	YOR322C
YER074W	YNR045W	YDR500C	YOR324C
YLR061W	YOL093W	YEL015W	YOR327C
YKL191W	YOR051C	YER049W	YPL009C
YML009C	YOL027C	YER153C	YPL013C
YMR143W	YOL114C	YFR001W	YPL127C
YOR182C	YOL039W	YGL049C	YPL197C
YDL037C	YPL081W	YGL070C	YPL203W
YIL133C	YOR252W	YGR010W	YPL226W
YDL184C	YOR242C	YGR011W	YPL239W
YMR129W	YOR354C	YGR012W	YPL240C
YDR502C	YPL208W	YGR014W	YPR014C
YPL106C	YPL119C	YGR015C	YPR028W
YDR116C	YOR276W	YGR016W	YPR057W
YKL068W	YPL009C	YGR017W	YPR075C
YLR325C	YOR303W	YGR018C	YPR076W
YLR398C	YGL049C	YGR019W	YPR077C
YOR312C	YFL001W	YGR021W	YPR078C
.YPR132W	YPR111W	YGR022C	YPR079W
YDL088C	YBR267W	YGR054W	YPR084W
YDL175C	YBR261C	YGR054W	YPR089W

YBR189W	YDL001W	YGR131W	YPR090W
YHR121W	YDL130W-A	YGR132C	YPR092W
YMR171C	YBR280C	YGR133W	YPR093C
YGR276C	YBR271W	YGR134W	YPR095C
YER091C	YCR021C	YGR135W	YPR100W
YAL029C	YDL048C	YGR136W	YPR152C
YBL087C	YCR059C	YGR137W	YPR197C
YDR312W	YCL011C	YGR138C	
YHR021C	YDR198C	YGR139W	
YKL167C	YDR333C	YGR141W	
YML026C	YDR207C	YGR142W	
YOR133W	YDR206W	YGR148C	
YPL198W	YDR101C	YHR010W	
YPR132W	YDR098C	YHR086W	
YFR031C-A	YDL210W	YHR087W	
YDL081C	YDR225W	YHR092C	
YDL160C	YDR117C	YHR093W	
YDL133C-A	YDL229W	YHR094C	
YNL087W	YDR001C	YHR095W	
YJL051W	YDR120C	YHR096C	
YJR137C	YDR257C	YHR097C	
YNL323W	YDR007W	YHR103W	
YBL072C	YDR156W	YHR104W	
YGL031C	YDR282C	YHR105W	
YHR203C	YDR169C	YHR202W	
YLR107W	YDR159W	YHR203C	
YOR017W	YER151C	YHR204W	
YBR048W	YEL011W	YHR206W	
YDL130W	YFL023W	YHR207C	
YDR471W	YFL034W	YHR209W	
YNL001W	YER007C-A	YHR210C	
YKR094C	YER002W	YIL001W	
YOL121C	YDR535C	YIL002C	
YLR434C	YER035W	YIL005W	
YDR363W	YGL208W	YIL006W	
YHR034C	YFR015C	YIL018W	
YGL222C	YGR053C	YIL074C	
YKL130C	YGR081C	YJL092W	
YMR188C	YGL173C	YJL106W	
YFR049W	YGL043W	YJL107C	
YGL147C	YHR047C	YJL117W	
YDL082W	YGR173W	YJL119C	
YDL136W	YHL034C	YJL120W	
YKL205W	YIR009W	YJL138C	
YMR190C	YJL131C	YJL151C	
YBL079W	YJR008W	YJL152W	
YIL079C	YJL122W	YJL153C	
YGR148C	YIL093C	YJL154C	
YLR048W	YJL141C	YJL155C	
YOR234C	YJL137C	YJL177W	
YOL041C	YIL110W	YJR034W	
YPR129W	YIL096C	YJR043C	
YJR047C	YJL148W	YJR047C	
YOL115W	YJL164C	YJR048W	

YLR448W	YJL160C	YJR049C
YJR148W	YJL158C	YJR050W
YDR382W	YJL187C	YJR066W
YGR214W	YIL162W	YKL003C
YKR026C	YKR058W	YKL127W
YNL302C	YJR126C	YKR036C
YPR042C	YKL109W	YKR039W
YIL071C	YKR024C	YKR040C
YIL103W	YKL139W	YKR041W
YPL183W-A	YKL166C	YKR042W
YKL001C	YLR343W	YKR043C
YDR385W	YLR221C	YKR044W
YGR072W	YLR003C	YKR045C
YKR057W	YLR262C-A	YKR046C
YLR367W	YLR258W	YKR047W
YNL301C	YLR137W	YKR048C
YPL220W	YLR150W	YLL028W
YOR302W	YLR149C	YLL029W
YIL148W	YLR289W	YLL032C
YDL061C	YMR186W	YLL038C
YER176W	YML007W	YLL039C
YOL137W	YML109W	YLL040C
YJL124C	YML028W	YLL041C
YCL009C	YMR139W	YLL042C
YBR057C	YNL141W	YLL043W
YJL176C	YNL255C	YLL044W
YMR307W	YNL031C	YLL045C
YEL048C	YMR247C	YLR296W
YJL095W	YNL300W	YLR297W
YKR020W	YMR282C	YLR299W
YNL171C	YMR273C	YLR300W
YOR115C	YNL197C	YLR303W
YPL069C	YNL307C	YLR306W
YOL006C	YNL081C	YLR307W
YOR028C	YMR297W	YLR309C
YPR030W	YNL229C	YLR311C
YBR030W	YNL227C	YLR312C
YBR026C	YNL224C	YLR313C
YBL024W	YNR048W	YLR401C
YBR034C	YOR035C	YLR402W
YAL040C	YOL001W	YLR404W
YBR064W	YOL114C	YLR405W
YBR062C	YOR083W	YLR406C
YBR186W	YOR078W	YLR407W
YBR185C	YOL031C	YLR408C
YBL104C	YPL052W	YLR410W
YBR222C	YPL157W	YLR412W
YBR010W	YPL067C	YLR413W
YBR009C	YPL184C	YLR414C
YBL003C	YBR232C	YLR441C
YCR071C	YBR240C	YML112W
